# An Approximate Bayesian Computation Approach for Modeling Genome Rearrangements

**DOI:** 10.1093/molbev/msac231

**Published:** 2022-10-25

**Authors:** Asher Moshe, Elya Wygoda, Noa Ecker, Gil Loewenthal, Oren Avram, Omer Israeli, Einat Hazkani-Covo, Itsik Pe’er, Tal Pupko

**Affiliations:** The Shmunis School of Biomedicine and Cancer Research, George S. Wise Faculty of Life Sciences, Tel Aviv University, Tel Aviv 69978, Israel; The Shmunis School of Biomedicine and Cancer Research, George S. Wise Faculty of Life Sciences, Tel Aviv University, Tel Aviv 69978, Israel; The Shmunis School of Biomedicine and Cancer Research, George S. Wise Faculty of Life Sciences, Tel Aviv University, Tel Aviv 69978, Israel; The Shmunis School of Biomedicine and Cancer Research, George S. Wise Faculty of Life Sciences, Tel Aviv University, Tel Aviv 69978, Israel; The Shmunis School of Biomedicine and Cancer Research, George S. Wise Faculty of Life Sciences, Tel Aviv University, Tel Aviv 69978, Israel; The Shmunis School of Biomedicine and Cancer Research, George S. Wise Faculty of Life Sciences, Tel Aviv University, Tel Aviv 69978, Israel; Department of Natural and Life Sciences, Open University of Israel, Ra'anana, Israel; Department of Computer Science, Columbia University, New York, USA; The Shmunis School of Biomedicine and Cancer Research, George S. Wise Faculty of Life Sciences, Tel Aviv University, Tel Aviv 69978, Israel

**Keywords:** genome rearrangement, approximate Bayesian computation, genome evolution

## Abstract

The inference of genome rearrangement events has been extensively studied, as they play a major role in molecular evolution. However, probabilistic evolutionary models that explicitly imitate the evolutionary dynamics of such events, as well as methods to infer model parameters, are yet to be fully utilized. Here, we developed a probabilistic approach to infer genome rearrangement rate parameters using an Approximate Bayesian Computation (ABC) framework. We developed two genome rearrangement models, a basic model, which accounts for genomic changes in gene order, and a more sophisticated one which also accounts for changes in chromosome number. We characterized the ABC inference accuracy using simulations and applied our methodology to both prokaryotic and eukaryotic empirical datasets. Knowledge of genome-rearrangement rates can help elucidate their role in evolution as well as help simulate genomes with evolutionary dynamics that reflect empirical genomes.

## Introduction

### Evolutionary Changes, Micro, and Macro-scale

Genomic evolutionary events can be roughly divided into gene-scale and genome-scale. Gene-scale events include substitutions of DNA bases and short insertions and deletions of DNA blocks, which mainly affect single genes. Genome-scale events include gene order rearrangements, changes in chromosome number, and even whole-genome duplications. Genome-scale events have been studied as early as 1938 with the study of gene inversion in *Drosophila* (Dobzhansky and Sturtevant 1938). Inferring the rates of genome-scale events across a phylogenetic tree can provide insights into various aspects of evolution. For example, it revealed a correlation between the rates of genome-scale events and the rate of speciation (Navarro and Barton 2003; Zhao et al. 2004; Mayrose et al. 2010).

### Previous Studies of Macro-scale Changes

Genome-scale events were mainly modeled and quantified using deterministic graph-based approaches. A great effort was directed toward developing efficient algorithms for inferring the minimal number of inversion or translocation events that explain the differences in genome rearrangements between two genomes using a measurement called breakpoint distance (Bryant 2000; Elias and Hartman 2006; Hartmann et al. 2018). Another variation on the breakpoint distance called “single cut or join” (SCJ) was developed and was shown to simplify the solutions to some of the harder problems in the subject of genome rearrangement, such as genome halving, aiming to reconstruct an ancestor of a genome that underwent a whole-genome duplication event, on a genome consisted of multiple linear chromosomes (Feijão and Meidanis 2011). The SCJ model was also used to infer the topology of the phylogenetic tree (Biller et al. 2013). Blin et al. (2004) computed breakpoint distance, which can be used for genomes with multiple genes from each gene family.

These methods do not rely on a probabilistic approach, and thus fail to benefit from the advantages of probabilistic evolutionary models, for example the ability to statistically test the fit of different hypotheses (models) to empirical data. In addition, they ignore the inherent stochasticity of the evolutionary process, thus they do not adequately account for inference uncertainty. Most probabilistic methodologies are based on the likelihood function. For example, when modeling substitutions events along a tree, it is straightforward to compute the likelihood as the multiplication of all alignment position likelihood scores. The use of multiplication is justified when positions are assumed to evolve independently of each other.

Ideally, genome-scale events would also be analyzed within the framework of probabilistic evolutionary models. The main challenge of using probabilistic methods to model genome-scale events is that one cannot assume that different genes evolve independently from each other, that is that each gene evolves under a separate Markov model. Some works did applied probabilistic methods to genome evolution, Larget et al. (2002) utilized the Markov Chain Monte Carlo (MCMC) approach to reconstruct the phylogenetic tree topology using mitochondrial gene order data. An MCMC approach was also used to estimate the number of inversion events between two chromosomes (York et al. 2002) or the number of inversions, translocations, and inverted translocations (Miklós 2003). Moreover, Larget et al. (2005) estimated ancestral gene orders, using a model in which the genome is composed of a single chromosome and rearrangements occur by inversions only. Miklós and Smith (2015) created a Gibbs sampler for sampling the most parsimonious genome assignments on the phylogenetic tree, under the aforementioned SCJ model. Another relevant probabilistic approach aimed to infer horizontal gene transfer events (Sevillya et al. 2020).

### Approximate Bayesian Computation

When analyzing genome rearrangement events, even formulating the likelihood function is a formidable task. Such cases, in which the likelihood is either hard or impossible to compute, motivated the application of likelihood-bypassing methods, such as the Approximate Bayesian Computation (ABC) framework (Sisson et al. 2018). The ABC approach is thoroughly described in various places (e.g., Beaumont 2010; Sisson et al. 2018). We briefly describe it here for self-containment. We assume a stochastic genome rearrangement model M which depends on a *p*-dimensional parameter vector *θ*. Given empirical genomic data (*y*_obs_), we aim to approximate the posterior distribution of *θ* using Monte Carlo integration, which requires sampling from the posterior. Theoretically, this can be achieved using methods such as importance sampling, Markov chain Monte Carlo (MCMC), and sequential Monte Carlo (Doucet et al. 2001; Del Moral et al. 2006; Brooks et al. 2011). However, those algorithms require a direct evaluation of the likelihood function $p(y_{\mathrm{obs}}|\theta )$, which may be infeasible. Hence, ABC, which is a likelihood-free approach that only requires the ability to simulate data under the likelihood model P(y|θ) is used. Put simply, the ABC approach aims to approximate the posterior distribution by simulating many datasets under the likelihood model $y_{\mathrm{sim}}∼P(y|\theta )$ and only retaining simulated datasets for which the simulated and the observed data are “close enough”. Specifically, we make use of a classic ABC method, which is based on the rejection sampling algorithm. Assuming a prior distribution over model parameters *π*, the algorithm performs the following pipeline iteratively until *N* samples are obtained: 1) A parameter *θ*_*i*_ is generated from a proposal density *g*(*θ*), with *g*(*θ*) > 0 if *π*(*θ*|*y*_obs_) > 0; 2) data *y*_*i*_ are simulated from $y_{i}\;∼\;P(y|\theta_{i})$; 3) A summary statistics vector is calculated *s*_*i*_ = *S*(*y*_*i*_); 4) The parameter *θ*_*i*_ is accepted with probability $\frac{K_{h}(\left|\left|s-s_{\mathrm{obs}}\right||\;\pi (\theta_{i}\right))}{Kg(\theta_{i})}$. *K*_*h*_ is a kernel function, which is a nonincreasing function of the distance between the observed and simulated summary statistics, commonly scaled between 0 and 1. Using the kernel function allows assigning higher sampling probabilities for closer simulations. The subscript *h* stands for the scale parameter of the kernel function, which controls the extent to which closer samples are favored over distant simulations, that is as *h* → 0 only simulations for which it holds that *s* = *s*_obs_ are accepted. The most common kernel function is the uniform kernel, which corresponds to a deterministic accept–reject threshold, that is *K*_*h*_(*u*) = *I*(*u*) < *h* where *I* is the indicator function, with *I*(*Z*) = 1 if *Z* is true and *I*(*Z*) = 0 otherwise. The parameter *K* is a constant satisfying $\geq K_{h}(0)\underset{\theta }{\max}\;\frac{\pi (\theta )}{g(\theta )}$, thus ensuring acceptance probabilities between 0 and 1.

Of note, the obtained samples are not drawn from the true posterior distribution, since a low dimension (non-sufficient) summary statistic *s* is used instead of *y*. Moreover, samples for which the corresponding summary statistics vector is different but close enough to the observed one are also accepted. The ABC posterior can be interpreted as a continuous mixture of posteriors $\pi_{\mathrm{ABC}}(\theta \;|s_{\mathrm{obs}})=\int \;\beta (s)\pi (\theta |s)ds$, in which the mixing weight is the conditional density of *s* given it was accepted in step 4 (Fearnhead and Prangle 2012). However, using representative summary statistics and requiring a relatively small distance between the observed and sampled summary statistics is expected to result in an overall good approximation.

In our implementation, we used *g*(*θ*) = *π*(*θ*) and a deterministic accept–reject decision at step 4, which can be regarded as using a uniform kernel density. Furthermore, to increase the efficiency of our approach in terms of running time, we predetermined the number of simulations we draw and used a dynamic distance threshold, that is the distance threshold was determined by the maximal distance within the closest *m* simulations. We optimize *m* by testing several options and choosing the one that gave the best results for simulated data (see Results).

### Problems With Existing Simulation Tools

Using ABC for the inference of genome-rearrangement events necessitates the simulation of genomes based on a probabilistic rearrangement model. Several genome simulation tools exist. Simulators such as ALF (Dalquen et al. 2012) generate the entire genome sequences of the extant species along an underlying phylogenetic tree. This makes the simulation time for a single dataset on the order of hours of CPU time (>4 h for 20 species of *Escherichia coli* with a genome size of 4,352 genes). The ABC approach requires an order of 10^5^ simulations and thus, inferring a single dataset using existing simulation tools will require decades of CPU time. In addition, the model assumed in our ABC approach differs from those assumed in simulators such as ALF (Dalquen et al. 2012). This motivated us to implement our own genome simulator.

In this study, we first introduce two continuous-time probabilistic models for genome rearrangement. The first assumes that each genome includes a single (linear) chromosome and allows for inversion and translocation events only. The second expands the first by allowing multiple chromosomes and accounting for chromosome fission and fusion events. We next describe the ABC approach we implemented to infer the parameters of these models. We optimize our approach, balancing between accuracy and running time. We further determine the inference accuracy of the resulting inference scheme using extensive simulations. Finally, we apply our method to empirical yeast and bacteria datasets. Our results provide a framework to study genome-rearrangement events accounting for the tree topology, the branch lengths, and the inherent stochasticity of the evolutionary process. Our methodology also provides means for more realistic simulations of genomic sequences.

## New Approach

### Models

#### Genome Minimalistic Format

**Fig. 1.**
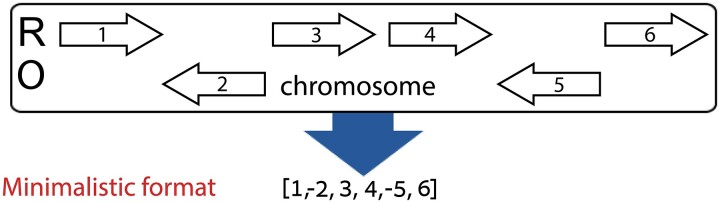
Translation of gene order to the minimalistic format. Shown is a chromosome in which genes are marked as arrows. R, reference strand; O, Other strand. Each gene is assigned with a unique number. In the minimalistic format, genes encoded on the other strand are marked with a minus sign. We note that as setting which strand is the reference strand is arbitrary, the above chromosome is equivalent to [−6, 5, −4, −3, 2, −1].

Our genome-rearrangement models use a minimalistic genome representation that captures the gene order and chromosome number information. In this format, each gene is assigned a unique natural number. Each chromosome is described by a list of genes and each genome is described by a set of chromosomes. Genes can reside on either strand of the chromosome. We consider one strand as the reference strand. Genes on the reference strands are assigned a positive number, while genes on the other strand are assigned a negative number (fig. 1). Reading direction for the chromosome (i.e., the decision on which strand is the reference) is arbitrary (inverting an entire chromosome would not change the results). In this work, for empirical datasets, the reference strand was determined based on the direction of the submitted genomic sequence data. It is treated as if it was arbitrarily chosen and thus does not affect the summary statistics. The input for our methodology is a set of genomes in this format, each corresponding to a unique species (or sub-species). Of note, orthologous genes on different genomes are assigned with the same number, but not necessarily the same sign. We also assume a phylogenetic tree that was reconstructed based on the core genome of the set. In this tree, branch lengths are measured as the expected number of nucleotide substitutions per site.

#### Basic Model

**Fig. 2.**
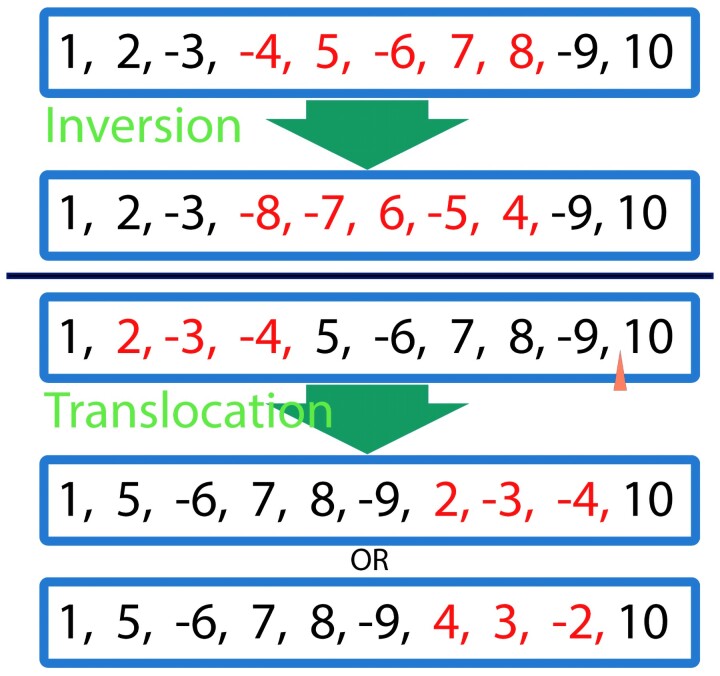
The two types of events that are accounted for in *M*_0_. Top: an inversion event with a block size of five. Bottom: a translocation event of size three with both orientation options for re-insertion. The genes that were involved in the event are marked in red.

In our basic model, *M*_0_, we assume a single chromosome in each genome and allow two types of events: 1) inversion, a reversal of a block of genes in its place; 2) translocation, a relocation of a block of genes (fig. 2). We note that there are no content-altering events, such as gene duplication or loss, and thus, the total genome content remains the same throughout evolution.

#### Advanced Model

Our advanced model, *M*_1_, extends the basic model by allowing genomes to contain multiple chromosomes. In this model, we also account for fusion and fission events, that is the merging of two chromosomes into one, and the splitting of a single chromosome into two, respectively. Under *M*_1_ genes can also be translocated from one chromosome to another.

#### Parameters

The set of parameters for all models considered in this study, as well as their assumed prior distributions, are given in table 1. Let *R*_in_ and *R*_tr_ denote the inversion and translocation rates, respectively. These rates determine the mean number of events per gene relative to the branch length, which are measured by the number of nucleotide substitutions per site (see Discussion regarding this choice). Both inversions and translocations operate on a block, namely, a sequence of adjacent genes. In our proposed models, we assume that the block size, that is the number of genes in a block, follows a trimmed Zipfian distribution, described using the exponent parameter *a* which characterizes the power law of the distribution, and a fixed value *m*_zip_ which is its maximal value: $f(k|a,m_{\mathrm{zip}})=\;\frac{k^{-a}}{\sum_{i=1}^{m_{\mathrm{zip}}}\;i^{-a}}$. In this work, the *m*_zip_ parameter is set to 50.

**Table 1.** Model Parameters Used. The *M*_0_ Model Assumes a Genome With a Single Chromosome, While the *M*_1_ Model Allows for Multiple Chromosomes and Fission and Fusion Events. a Prior Uniform Distribution (Unif) is Assumed for all Model Parameters.

Parameter	Model		Default prior
	*M*_0_	*M*_1_	
*a*-parameter (*a*)	✔	✔	Unif (1.001, 2.0)
Inversion rate (*R*_in_)	✔	✔	Unif (0, 1.5)
Translocation rate (*R*_tr_)	✔	✔	Unif (0, 1.5)
Number of chromosomes in the root (*ch*_root_)		✔	Unif (4, 18)
Fusion rate (*R*_fu_)		✔	Unif (0, 0.8)
Fission rate (*R*_*fi*_)		✔	Unif (0, 0.03)

As our models do not allow for events that change the number of genes along the tree, the total number of genes in the root *n*_root_, equals to the number of shared genes among extant species. Hence, *n*_root_ is not a free parameter of the model, but rather determined by the input dataset.

The *M*_1_ model extends the *M*_0_ model by allowing genomes to be composed of several chromosomes and by adding events that change the number of chromosomes in the genome. Thus, the model contains three additional parameters. Two of these are rate parameters for chromosome fusion and fission events, *R*_fu_ and *R*_fi_, respectively. Similar to *R*_in_ and *R*_tr_, *R*_fi_ is also measured per gene per unit of branch length. *R*_fu_ is measured per chromosome per unit of branch length. The model justification to normalize *R*_fu_ per chromosome rather than per gene is that while a fission event occurs as a result of a break in the DNA, and therefore should depend on the number of genes, fusion events occur as a result of fusion between two chromosome edges, and hence should correlate with the number of chromosome edges. Therefore, *R*_fu_ should be relative to the number of chromosomes. In addition to *R*_fu_ and *R*_fi_, as the chromosome number changes throughout the evolution, there is a need for another parameter, which is the number of chromosomes at the root, *ch*_root_.

#### Simulation

In the case of *M*_0_, given a set of model parameters {*R*_in_, *R*_tr_, *a*}, the total number of genes, *n*_root_, and a phylogenetic tree we simulate a set of genomes for the extant species of the tree as follows: We first generate a root genome, which is represented in the minimalistic format as a vector of the integers 1 to *n*_root_. Inversion and translocation events are then simulated along the tree with their respective rates. For each event, a starting location (the position from which the event block starts) is sampled uniformly. The event block size is then generated from the Zipfian distribution with the restriction that it must not exceed the end of the chromosome (if it exceeds this limit, another value is drawn from the Zipfian distribution). For translocation events, a destination location, which is the re-insertion point for the block, is drawn uniformly from the entire range of the genome excluding the block area (the range between the event location and the event location + block size, which is undergoing the translocation). The exclusion is needed to prevent a null event, that is an event that does not change the genome. In translocation events, we assume equal probabilities for the block to be reinserted either in its original orientation or inverted.

In the case of the *M*_1_ model, the set of parameters is {*R*_in_, *R*_tr_, *a*, *n*_root_, *ch*_root_, *R*_fu_, *R*_fi_}. We set the size of each of the *ch*_root_ chromosomes by giving each gene an equal probability to be found in each chromosome. To prevent cases in which some of the chromosomes are empty, we first assign one gene per chromosome before drawing chromosome locations for the rest. This results in a chromosome size distribution that is close to multinomial (in a multinomial distribution, a chromosome can have zero genes, while here, each chromosome has at least one gene). The generation of gene order and orientations is performed as described for *M*_0_. Both inversion and translocation events are also generated as described for *M*_0_. For fission events, a location is drawn uniformly from the genome with the exception that it cannot be at the ends of the chromosomes as it will result in a null event. The chromosome is then broken into two at the drawn location. For fusion events, two chromosomes are chosen with equal probabilities and one of the ends of the second chromosome is attached to one of the ends of the first chromosome with equal probability for each end.

#### Summary Statistics

We define the concept of a *unique block* (*UB*) as an identical gene sequence that is shared by at least two genomes in the same or inverted orientation, for example [1,2,3] along one genome and either [1, 2, 3] or [−3, −2, −1] along the other. We only consider maximal blocks, that is when comparing two genomes *G*_*i*_ and *G*_*j*_, we ignore a block *b* if it is contained in a longer block *b*′. The shorter block *b* can still be considered as part of the overall *UB*s (over all pairs), if *b* was found when comparing *G*_*a*_ with a different genome in the dataset, *G*_*k*_, but *b*′ was not found in that comparison. We call these *blocks unique* because, if a block is shared by multiple genomes, we only count it once. We also define a *probably inverted unique block* (*PIUB*), which is defined as a *UB* that is adjacent to at least one common neighbor in a reversed orientation. For example, the block [2, 3] is a *PIUB* for the genomes [1, 2, 3, 4] and [1, −3, −2, 5]. The existence of a *PIUB* gives a strong notion of the occurrence of an inversion event, as the probability to create a *PIUB* using other types of events (translocations, fissions, or fusion) is insignificantly small for any interesting data, as it is inversely correlated with the genome size.

To compute *UB*s and *PIUB*s we perform an “all-versus-all” comparison, comparing each pair of extant genomes. For a dataset of *L* genomes, this results in *O*(*L*^2^) comparisons (we further elaborate on it below, when discussing the complexity).

We note that both *UB*s and *PIUB*s share qualities with breakpoints, a concept widely used for computing distances between two genomes (Kaplan et al. 2000; Blin et al. 2004; Bafna and Pevzner 2005). Breakpoint distance was designed to measure the distance between just two genomes. Here our goal was to infer rearrangement events from multiple genomes, thus we use the related concept of *UB*s. Although one could measure breakpoint distances between each pair of genomes in the data, the use of *UB*s reduces multiple counts of the same events. For example, a *UB* that is shared among three genomes will be counted once, while a breakpoint may be counted more than once when all three pairwise genome comparisons are computed. In addition, the concept of *UB* is more directly related to the size of the event, which is explicitly modeled in this work, than the breakpoint distances are. Furthermore, accounting for *PIUB*s can help differentiate inversion from translocation events. Finally, we note that there is a linear relationship between breakpoint distance and the number of inferable inversions and translocations when two chromosomes are analyzed. Specifically,

Br≤2Inv+3Trans

Where *Br*, *Inv*, and *Trans* are the number of breakpoints, inversions, and translocations, respectively, as an inversion events created by breaking the sequence on both sides of the inverted block, resulting in two breakpoints, while a translocation event, breaks the sequence in three places resulting in three breakpoints (Miklós 2003; Bafna and Pevzner 2005). The inequality is tight when each event is using its unique breakpoints. Similarly, there is a linear relationship between the sum of all *UB*s in a comparison between two genomes and the number of inversion and translocation events which is,

UB≤2Inv+3Trans+1

In fact, the relationship between *UB*s and breakpoints is,

UB=Br+1

We note that these equations are approximations that hold for most evolutionary scenarios. For example, events that occur at the edges of a chromosome would result in one less *UB* and breakpoint. Furthermore, both *Br* and *UB* account for inferable events, that is events that can easily be identified. Some events can be either untraceable or misidentified. For example, two consecutive inversions in the exact same location will leave the genome unchanged and thus, would not change the number of *UB*s. In this case, the above equation connecting the number of inversions and translocations to the number of *UB*s will not hold. We assume that these types of events are rare and that these equations do point to the strong dependence between *UB* and *BR*.

When comparing a pair of genomes, the summary statistics for the *M*_0_ model are the counts of *UB*s of each size ranging from 1 to a maximum size, *S*_bl_ (by default we set *S*_bl_ to 10, but this can be modified by the user) as well as *PIUB*s of the same range. We also use an overflow bin for each block type to collect all blocks longer than *S*_bl_. The total number of summary statistics is, therefore, 2*S*_bl_ +2 (22 for the default *S*_bl_).

**Fig. 3.**
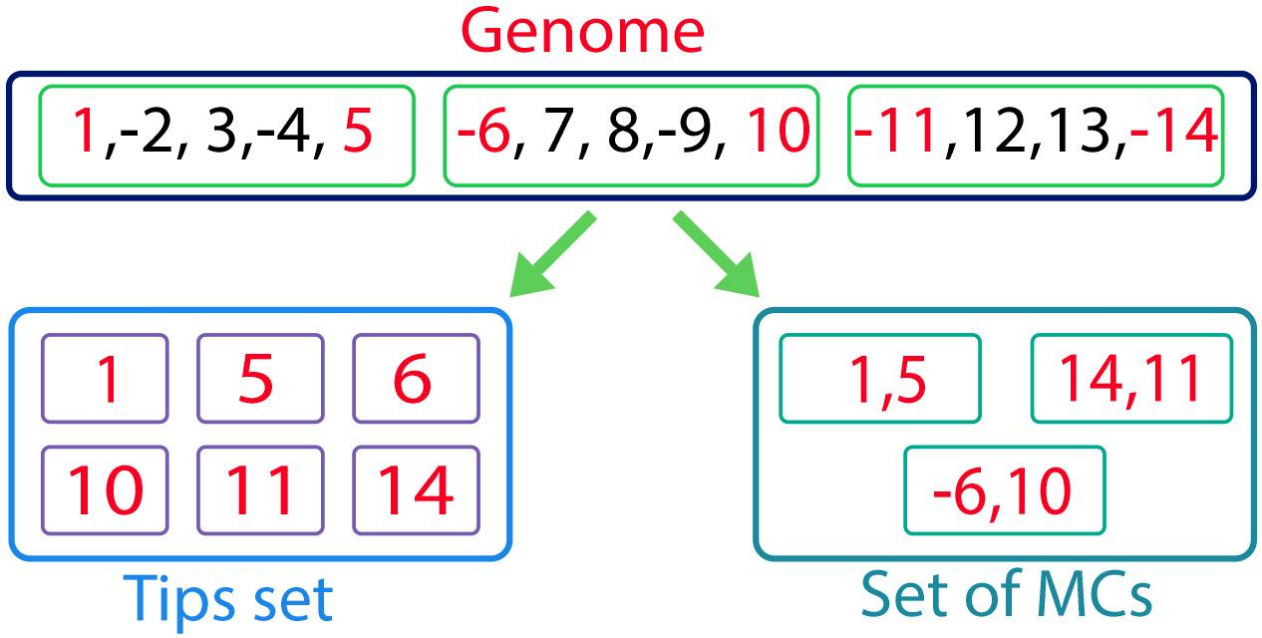
Illustration of the relations between a genome and its corresponding tips set and set of minimalized chromosomes (*MC*s). In this genome, there are three chromosomes. Each beginning or end of a chromosome is called a tip (in red). The tips set is just the collection of all tips regardless of their orientation. In contrast, the *MC* is an ordered pair that includes the tips of a chromosome, with their corresponding orientation.

In the *M*_1_model, we introduce an additional set of summary statistics. First, we introduce the following four summary statistics: minimum, maximum, mean, and variance of chromosome numbers in the leaf genomes. Additional features are based on the following definitions (fig. 3): 1) A *tip* is either the first or last gene of a given chromosome. 2) Given a chromosome, a *minimalized chromosome* (*MC*) is an ordered couple of both tips of the chromosome with their orientation, thus, each chromosome is associated with an *MC* and, consequently, each genome can be associated with a set of *MC*s. While we account for the orientation of the genes in each *MC*, the orientation of the entire chromosome is arbitrary (see above). Thus, the *MC* [a, b] is equivalent to the *MC* [−b, −a] but not to the *MC* [−a, b]. 3) A *tip set* is the set of all tips in the set of *MC*s, disregarding their orientation (see fig. 3). Intuitively, differences in the sets of *MC*s between descendants of a node in a phylogenetic tree are highly informative for detecting fission and fusion events (we note that translocations and inversions at end of chromosomes also affect *MC*s). To allow inference for internal nodes for which genomic data are not available, we associate each internal node of the phylogenetic tree with a set of *MC*s (and hence also a set of tips), which is the union of the sets of *MC*s of its descendant nodes.

**Fig. 4.**
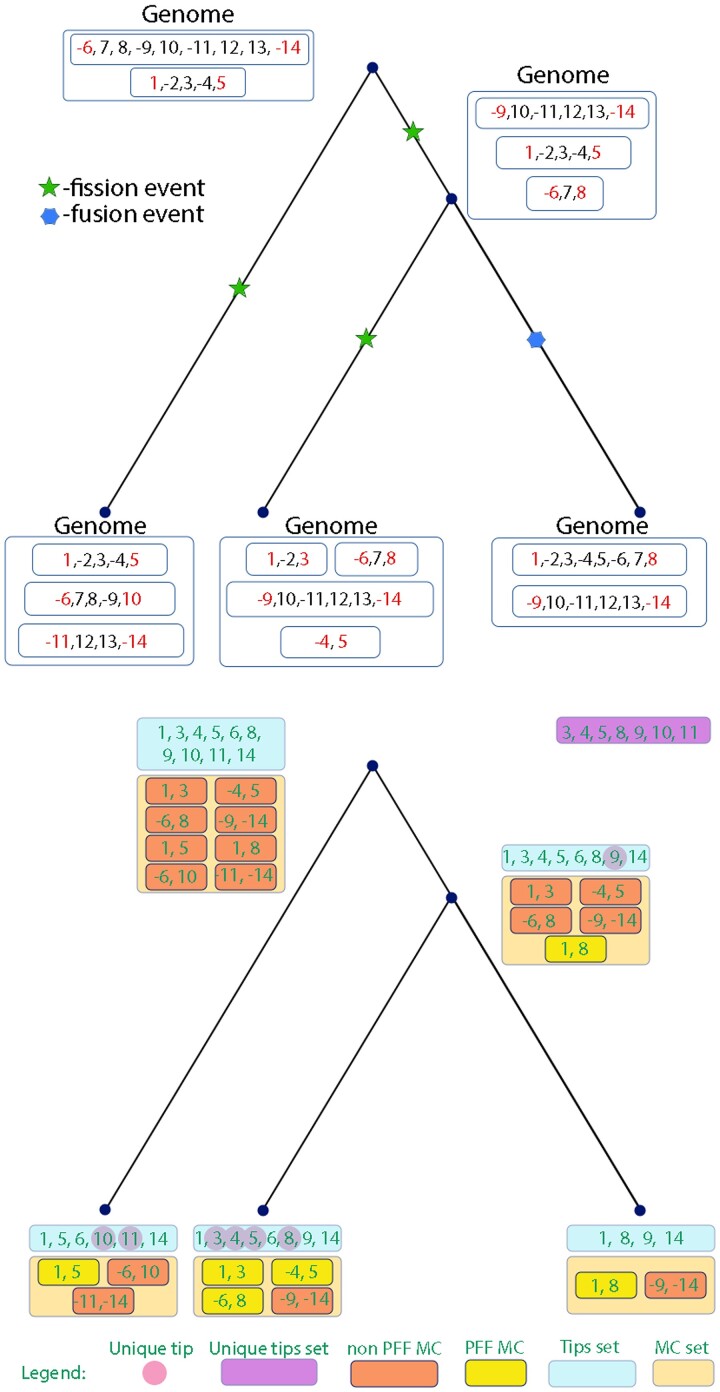
Calculation example for *M*_1_ exclusive features. Above: an example for chromosome evolution with three fission events and one fusion event. Below: calculation of summary statistics for the above data. *MC*s that correspond to *PFF* events are marked in yellow. Unique tips are embedded in a pink circle in the descendant node where they were first identified as such. The summary statistics set is [2, 4, 3, 1.0, 7, 6, 10, 8] for the following features: minimum, maximum, mean, and variance of chromosome numbers, the size of the unique tip set in the tree, the total number of *PFF* events in the tree and the sizes of the tip set and the minimalized chromosome set in the root, respectively.

Counting sizes of tip sets and minimalized chromosomes mapped onto the phylogenetic tree may provide valuable information regarding fission and fusion events. We add three summary statistics that are based on tips. An example for the extraction of these features is given in fig. 4. Specifically, two additional summary statistics that we use are: 1) the size of the tip set at the root; 2) the size of the set of *MC*s reconstructed at the root. We additionally define the term unique tip. Given a node, a *unique tip* is a tip that can be found in the tip set of a node and not in the tip sets of its sibling nodes. Intuitively, this implies a fission event on the branch leading to this son or a fusion event in the branch leading to its sibling (or siblings for multifurcating nodes). We define a unique tip set as the set of all unique tips in the tree. This set includes the union over all unique tips along the tree. Based on this definition, we also include the size of this group as an additional summary statistic (see table 2 for a full list).

**Table 2.** List of Summary Statistics.

#	Description	Model
1	Number of *UBs* of size 1	*M*_0_
2	Number of *UBs* of size 2	*M*_0_
3	Number of *UBs* of size 3	*M*_0_
4	Number of *UBs* of size 4	*M*_0_
5	Number of *UBs* of size 5	*M*_0_
6	Number of *UBs* of size 6	*M*_0_
7	Number of *UBs* of size 7	*M*_0_
8	Number of *UBs* of size 8	*M*_0_
9	Number of *UBs* of size 9	*M*_0_
10	Number of *UBs* of size 10	*M*_0_
11	Number of *UBs* larger than 10	*M*_0_
12	Number of *PIUBs* of size 1	*M*_0_
13	Number of *PIUBs* of size 2	*M*_0_
14	Number of *PIUBs* of size 3	*M*_0_
15	Number of *PIUBs* of size 4	*M*_0_
16	Number of *PIUBs* of size 5	*M*_0_
17	Number of *PIUBs* of size 6	*M*_0_
18	Number of *PIUBs* of size 7	*M*_0_
19	Number of *PIUBs* of size 8	*M*_0_
20	Number of *PIUBs* of size 9	*M*_0_
21	Number of *PIUBs* of size 10	*M*_0_
22	Number of *PIUBs* larger than 10	*M*_0_
23	Minimum chromosome number in a leaf	*M*_1_
24	Maximum chromosome number in a leaf	*M*_1_
25	Mean chromosome number in the leaves	*M*_1_
26	Variance of chromosome number in the leaves	*M*_1_
27	Size of set of *MC*s in the root	*M*_1_
28	Size of tip set at the root	*M*_1_
29	Number of unique tips	*M*_1_
30	Number of *PFF*s	*M*_1_

We define a *probable fission-fusion* (*PFF*) event as follows: to be considered as a *PFF*, an *MC* [A, B] in an internal node must appear in the set of *MC*s that corresponds to the node, that is, it appears in the set of *MC*s in at least one son, and there is at least one other son for which [A, B] is absent in the set of its *MC*s. We emphasize that when requesting that an *MC* [A, B] is absent, we request that it is absent in both orientations: [A, B] and [-B, -A]. We further request that both A and B appear in the tips set of that node for which [A, B] is absent. Intuitively, this means that either [A, B] was present in the node and underwent a fission event along the branch leading to its descendant node, or [A, X] and [Y, B] were present in the node and a fusion event led to [A, B] (supplementary fig. S1, Supplementary Material online). The total number of *PFF* events along the tree is an additional summary statistic that we use.

#### Unique blocks Algorithm

We start by describing an algorithm for finding *UB*s and *PIUB*s between two genomes, *G*_1_ and *G*_2_ (each of which may be composed of several chromosomes).

##### Preprocessing

We assume that the chromosomes in each genome are arbitrarily numbered (1, 2, …). As stated above, within each chromosome the genes are numbered according to the minimalist format. For a genome *G,* we create a data structure that enables quick retrieval of gene location. Given a gene *g*, we maintain *sign*_*G*_(*g*), *chr*_*G*_(*g*), and *location*_*G*_(*g*), which correspond to the orientation of the gene, the chromosome in which it is located, and the gene location within the chromosome, respectively. For example, for genome *G*, if the second gene in the seventh chromosome is 5 and its orientation is reversed (i.e., −5) then *sign_G_*(5) = − 1, *chr_G_*(5) = 7 and *location_G_*(5) = 2. Also, for convenience, we assume that these methods disregard the sign of input *g*. Hence, for the example above we have *location_G_*(5) = *location_G_*(− 5) = 2.

###### Algorithm

####### Unique blocks

The algorithm is illustrated in supplementary fig. S2, Supplementary Material online. Let “current block” (*CB*) be a list of genes, which is initialized to be empty. We go over *G*_1_ in order, from the first gene in the first chromosome to the last gene in the last chromosome (to better illustrate the algorithm, we assume that a chromosome is an ordered list from left to right, and thus we scan *G*_1_ from left to right). For each gene *g* in *G*_1_, we first append it to *CB*. If *g* is the last gene in the chromosome, *CB* cannot be extended any further, and therefore we add *CB* to the set of *UB*s (see description below) and move to the next chromosome, resetting *CB*. If *g* is not the last gene in the current chromosome, we find the location of *g* in *G*_2_ using $\mathrm{ch}r_{G_{2}}(g)$ and $\mathrm{locatio}n_{G_{2}}(g)$. Our goal is to test whether we can extend the match between *G*_1_ and *G*_2_ to the next gene. Let *n*_1_ be the next gene in *G*_1_. We define direction *d* as $\mathrm{sig}n_{G_{1}}(g)\times \mathrm{sig}n_{G_{2}}(g)$. If *d* is positive, we search the next gene in *G*_2_ to the right of gene *g* in *G*_2_, while if *d* is negative, we search the next gene to the left of gene *g*. Let *n*_2_ be the next gene in *G*_2_, accounting for the direction *d*. Formally, we assign to *n*_2_ the gene in *G*_2_ which is in chromosome $\mathrm{ch}r_{G_{2}}(g)$ and in location, $\mathrm{locatio}n_{G_{2}}(g)+d$. If we pass the chromosome edge in *G*_2_ or if *n*_1_ is not equal to *n*_2_ × *d*, we end the block, add *CB* to the set, and reset it. If *n*_1_ equals *n*_2_ × *d*, we continue the current block. See supplementary fig. S2, Supplementary Material online for an example.

####### Probably inverted unique blocks

We first note that by definition, every *PIUB* is also a *UB*. Therefore, upon finding a *UB*, we check its immediate neighbors to determine if it is also a *PIUB*. Given the order [a, B, c] in *G*_1_ where a and c are single genes and B is a *UB*, B is also a *PIUB* if it is in *G*_2_ in one of the following: [x, −B, c], [−c, B, x], [a, −B, x], or [x, B, −a] (where the “x” marks an arbitrary gene). We note that this definition also includes cases in which both immediate neighbors surrounding B are shared between *G*_1_ and *G*_2_, for example [a, −B, c].

####### Algorithm complexity

The time-consuming step of this inference scheme is the computation of *UB*s and *PIUB*s for each simulated dataset. To allow quick retrieval of gene locations, needed for the computation of *UB*s and *PIUB*s, we use an array for each genome *G*, in which position *i* in the array corresponds to the location of gene *i*. This allows us to access location and orientation in time complexity of *O*(1). Creating such an array requires *O*(*n*_root_) both in terms of memory and running time. Therefore, for data with *L* extant species, the overall time and memory complexity of this preprocessing stage is *O*(*n*_root_ × *L*). Next, the pairwise algorithm described for finding *UB*s and *PIUB*s has a time complexity of *O*(*n*_root_). The justification for this complexity is that for each gene in *G*_1_, we find its counterpart in *G*_2_ in *O*(1). Comparing the next genes in both genomes is also *O*(1), as explained above. Regardless of whether the next gene *G*_1_ matches the next gene in *G*_2_ or not, we move to the next gene in *G_1_*, thus for each gene in *G*_1_, we perform *O*(1) operations. As the number of genome comparisons we perform is bounded by the number of nodes in the tree, which is also *O*(*L*), the total complexity of the *UB* and *PIUB* calculation is *O*(*n*_root_ × *L*). Thus, both the preprocessing stage and the computation is *O*(*n*_root_ × *L*). We note that time complexity analysis holds for both the *M*_0_ and *M*_1_ models (*M*_0_ can be viewed as a special case of *M*_1_, in which the number of chromosomes is 1).

### Parameter Inference

The input to our algorithm is a set of genomes in the aforementioned minimalistic format and a phylogenetic tree describing the evolutionary relationship among these genomes. We assume a uniform prior distribution over all model parameters (table 1). The effect of this assumption is later tested (see Results). The algorithm starts by calculating the set of summary statistics for the given input genomic data. Next, a large number of datasets is simulated (*s*_abc_) by drawing random rearrangement events along the input phylogenetic tree, assuming parameters drawn from the prior distributions. After each simulation, a vector of summary statistics is estimated and then compared to the summary statistics vector derived from the input data. This comparison is based on a weighted Euclidian distance between the summary statistic vectors, such that the weight assigned for each dimension is the standard deviation of the corresponding summary statistic across all simulations. Then, out of the *s*_abc_ simulations, only a small number (*m*) of closest simulations is kept. According to the ABC theory, the empirical distribution of the parameters derived from the closest simulations should provide a reasonable approximation for the true posterior distribution (henceforth for brevity, we use “posterior” to discuss the approximated posterior, and “true posterior” to discuss the real posterior). Here, we use the mean of this posterior distribution as our point estimate for each parameter.

In *M*_1_ we infer the parameters inherited from *M*_0_ using the same summary statistic set as the one used in *M*_0­_. The *M*_1_ unique parameters are inferred only with the set of summary statistics introduced for this model. This separation is due to the low relevance of the *M*_0­_ summary statistics to the *M*_1_ parameters and vice versa.

### ABC Tunable Parameters

The ABC inference accuracy depends on the total number of simulations (*s*_*abc*_) and the number of simulations retained (*m*). Setting high values for *s*_*abc*_ may increase accuracy by supplying more simulations, and thus potentially finding more simulated datasets with sampled summary statistics that resemble the input empirical dataset. However, *s*_*abc*_ is limited by the available computational resources. In contrast, the value of *m* does not affect running time. Low *m* values result in a higher similarity between the set of retained samples and the empirical dataset. However, the inference is then based on a small-sized sample, resulting in potential high sampling variance. Conversely, large *m* values result in the inclusion of samples with a higher distance from the empirical dataset, nevertheless reducing the sampling variance. While the values of *s*_*abc*_ and *m* can be provided as input, below we optimize *m* using a predetermined *s*_*abc*_.

### Measuring Performance

We quantified the inference accuracy using the coefficient of determination (*r*^2^, the square of Pearson's *r*) between the inferred and true parameters, which were used to generate the data. All model optimizations were performed using this measure of accuracy. We additionally quantified the accuracy using the Mean Squared Error (MSE), which showed the same trend as the *r*^2^ (see Results).

## Results

### Feature Selection

As the number of summary statistics increases, it is harder to find simulations that generate summary statistics that are “close” to those obtained from the data analyzed. Thus, reducing the dimension of the summary statistics may raise the estimation accuracy. It also provides insights into which summary statistics are important for inference. To test whether some of the summary statistics used can be discarded, we used the “leave-one-out” method, in which we compared the accuracy of the ABC method with all summary statistics, against variants of the ABC method, wherein each such variant, one of the summary statistics is not used. The summary statistic whose removal increases the *r*^2^ most is removed, and the process is iteratively repeated until no significant increase (<0.01) in performance is observed. For most parameters, the benefit of such a feature selection approach was not substantial, and we hence base our inference of these model parameters on the entire set of summary statistics (not shown). However, for the *a* parameter, the parameter that controls the number of genes involved in a rearrangement event, we managed to improve the accuracy from *MSE* = 0.018 (*r*^2^ = 0.78) using all features, to *MSE* = 0.016 (*r*^2^ = 0.82) when excluding summary statistics 1, 3, 4, 5, 6, 7, 9 (table 2). We additionally studied the inference accuracy when the inference is based on a single summary statistic. Such an analysis can help elucidate which single summary statistics harbors the most relevant information for the inference of each parameter, without accounting for the effect of the other summary statistics. It can also quantify the contribution of combining many summary statistics instead of using a single summary statistic. The results in supplementary table S2, Supplementary Material online clearly show that combining several summary statistics substantially contributed to the inference accuracy. For example, while the *MSE* for inferring the translocation rate when using all summary statistics was 0.005, the *MSE* relying on a single feature varied from 0.0271 to 0.116. For this specific parameter, the lowest *MSE* was obtained for the summary statistics measuring the number of *PIUB*s of size 4, although similar accuracy was obtained for the summary statistics number of *PIUB*s of size 3 and 5. For the *M*_1_ model, the three summary statistics that contributed most for the inference of the number of chromosomes in the root were the minimum, maximum, and variance of the number of chromosomes in the leaves (supplementary table S2, Supplementary Material online).

### Optimization Results

The ABC methodology relies on repeated simulations of datasets and the selection of a subset of these simulations whose summary statistics are relatively close to those of the empirical dataset analyzed. Under the assumption that the smaller the distance between the simulations and the data, the better the ABC posterior distribution approximates the true posterior distribution, using larger *s*_*abc*_ value is expected to improve the inference accuracy. As the simulation and extraction of summary statistics are the time-consuming part of the ABC scheme, we set *s*_*abc*_ = 200,000 to limit the running time to a manageable range. To optimize the accuracy of our inference scheme, we aim to find *m*, the optimal number of simulations used in the inference process (the above feature selection results were obtained with *m* = 200). We tested *m* values in the range of 1–2,000. Specifically, we generated 200 datasets with parameters drawn from the prior. We assumed a genome size (*s*_root_) of 6,350 genes and simulated datasets along the tree of yeast species, from 18 genomes (see Methods). For each of these datasets, for each choice of *m,* we inferred the model parameters. For each option of *m* and each model parameter, we computed the *r*^2^ and the *MSE* between the inferred and true parameters. Based on the performance with the various *m* values, we selected the *m* values for which the lowest *r*^2^ of all parameters was highest. The value chosen was *m* = 50 (see supplementary table S1, Supplementary Material online).

### *M*_0_ Model

#### Simulation-based Results

**Fig. 5.**
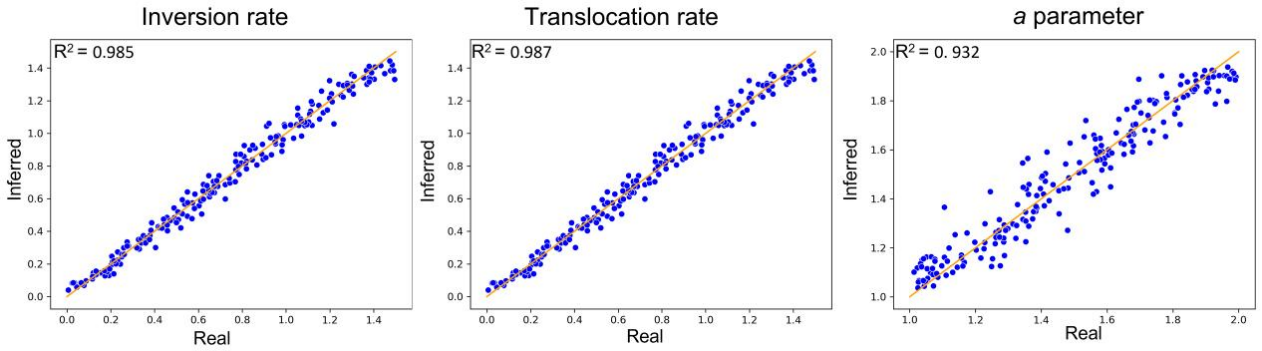
Scatter plots of the inferred (*Y*-axis) versus the parameters used for *M*_0_ simulation (*X*-axis). From left to right: inversion, translocation, and *a*-parameter. Each dot corresponds to a single simulation (out of a total of 200 simulations per plot). The line is the identity function *y* = *x*.

To quantify the accuracy of the ABC inference scheme, we simulated 200 datasets based on the bacterial phylogeny (see Methods). We then inferred the model parameters using ABC and computed *r*^2^ and *MSE* between the true parameters and the inferred ones for the chosen *s*_*abc*_ and *m* values. Very high values were observed for all parameters, *r*^2^ of 0.985, 0.987, and 0.932 and *MSE* scores of 0.00254, 0.00223, and 0.00568, for inversion rate, translocation rate, and *a*-parameter, respectively (fig. 5).

#### Effects of Genome Size and Branch Lengths on Inference

Although the branch lengths of the phylogenetic tree and the total number of genes (*n*_root_) are both factors that are intrinsic to the input data, we suspected that they could affect our ability to infer our parameters. Thus, we next studied how these factors affect inference accuracy. The total number of rearrangement events increases, on average, as a function of genome size, since the rates are calculated per gene. We, therefore, hypothesized that such an increase would provide more signal in the data and will enable more reliable estimation of model parameters. To test this hypothesis, we performed simulations using the following *n*_root_ options: 250, 500, 1,000, 2,500, 5,000, 10,000. For each option, we quantified accuracy based on simulations repeating the procedure described above. Our results clearly show that the accuracy increases as a function of genome size (table 3).

**Table 3.** Large Genome Size Increases Parameter-inference Accuracy. Accuracy is Measured as *r*^2^, Which Quantifies the Correlation Between True and Inferred Parameters, and *MSE*. Simulations Were based on the Phylogenetic Tree of the Bacterial Dataset (Model *M*_0_).

Genome size	*r* ^2^			*MSE*		
	Inversion	translocation	*a*-param	Inversion	translocation	*a*-param
250	0.798	0.86	0.547	0.0356	0.024	0.04
500	0.884	0.916	0.61	0.0194	0.0157	0.0312
1,000	0.926	0.952	0.75	0.0123	0.00897	0.02
2,500	0.967	0.98	0.883	0.0055	0.00384	0.0094
5,000	0.985	0.987	0.932	0.00254	0.00223	0.00568
10,000	0.99	0.993	0.96	0.0015	0.0013	0.00314

As sequences become more diverged, we expect an increase in the total number of rearrangement events. However, as sequences become more diverged, the reconstruction of rearrangement events becomes less reliable. We next studied how divergence levels affect inference accuracy. To this end, we quantified estimation accuracy as a function of total genome divergence. Specifically, we modified the level of divergence by changing the sum of branch lengths in the input phylogenetic tree. This is because we defined the rates relative to the number of substitutions, thus a longer branch length implies more genomic events. Branch lengths were multiplied by each of the following factors: 0.05, 0.1, 0.5, 1, 2, 5, 10. A strong positive correlation between divergence levels and inference accuracy was observed (table 4).

**Table 4.** Longer Branch Lengths are Associated With Increased Parameter-inference Accuracy. Accuracy is Measured as *r*^2^ and *MSE* Between the Inferred and True Values. The Input Trees Were based on the Bacterial Dataset Tree With Branch Lengths Multiplied by a Factor. Simulations are based on a Genome Length of 5,000 Genes (Model *M*_0_).

Branch length factor	*r* ^2^			*MSE*		
	Inversion	Translocation	*a*-parameter	Inversion	Translocation	*a*-parameter
0.05	0.834	0.876	0.53	0.0293	0.0214	0.0415
0.1	0.906	0.941	0.6	0.0157	0.0111	0.032
0.5	0.97	0.978	0.879	0.00508	0.00406	0.0097
1	0. 985	0. 987	0. 932	0.00254	0.00223	0.00568
2	0.991	0.991	0.952	0.00147	0.00162	0.0038
5	0.995	0.994	0.967	0.00079	0.00094	0.0026
10	0.996	0.996	0.976	0.000709	0.000708	0.0018

#### Bacterial Dataset Results

**Fig. 6.**
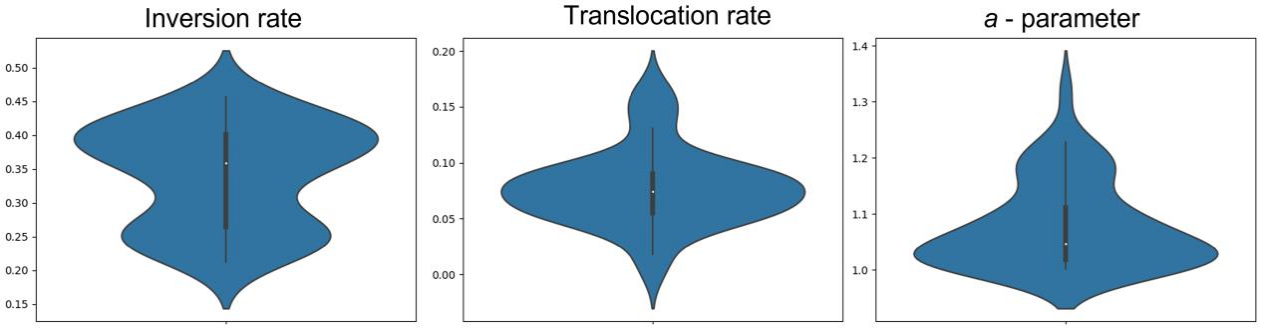
Violin plots of the 50 closest simulations for the microbial data inference. Note that, although the prior was distributed uniformly in the ranges seen in the graphs, the posterior is clearly centered around a much smaller range of values.

We next aimed to infer parameters for empirical data. To demonstrate the applicability of the *M*_0_ model, we selected bacterial genomes harboring a single chromosome (we regarded the chromosome as linear, as it appears in public datasets). We analyzed microbial genomes of 73 strains of *Escherichia* (Avram et al. 2019). We used the minimalistic format of the core genomes with *n*_root_ of size 1,875. We used the *s*_abc_ and *m* for each parameter as chosen in the optimization stage. The results were, 0.342, 0.078, and 1.08 for *R*_in_, *R*_tr_, and *a*-parameter, respectively. These results suggest that for these data, the inversion rate is about twice the rate of translocation and that the average block size involved in a rearrangement event is 9.88. We also show the violin plot of the closest 50 simulations to demonstrate the approximated ABC posterior distribution of the parameters (fig. 6).

### *M*_1_ Model

#### Simulation-based Results

**Fig. 7.**
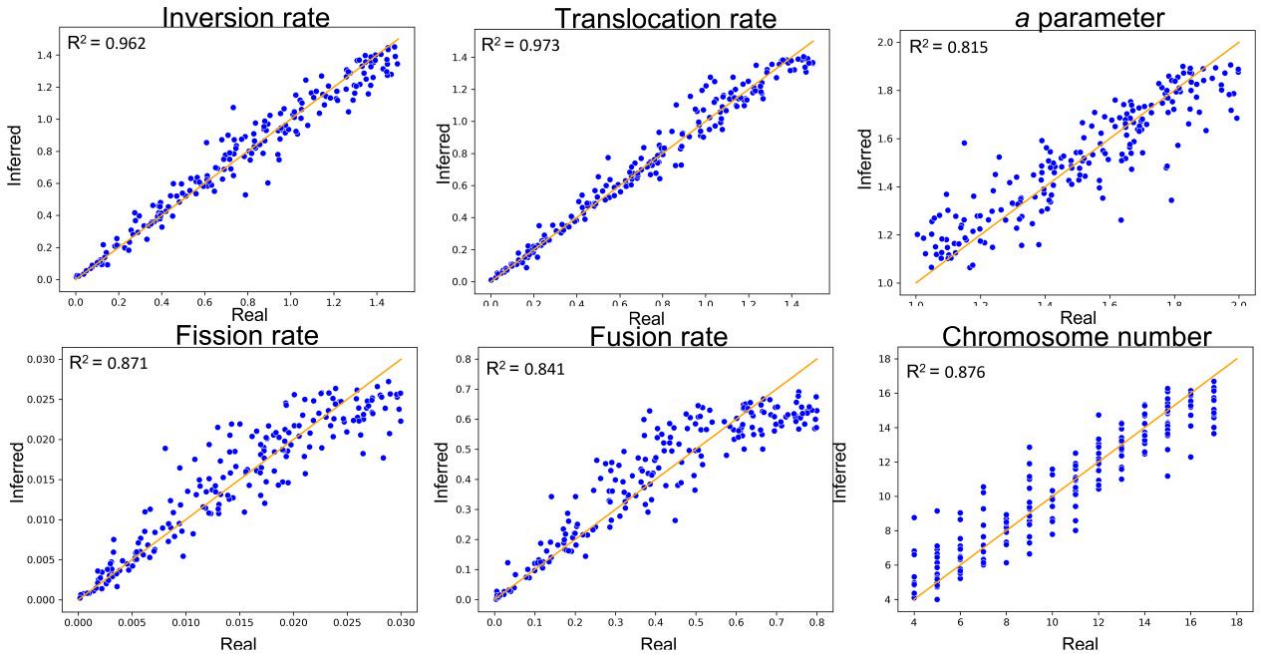
Scatter plots of the inferred parameters (inversion, translocation, *a*-parameter, fusion, fission, and chromosome number) versus the parameters used for *M*_1_ simulation. Each graph is based on 200 simulations. The green line is the identity *y* = *x* line.

We tested the *M*_1_ model using a tree of 18 *Candida* species based on data from CGOB (Maguire et al. 2013) and a genome size of 6,350 genes. We simulated 200 datasets with parameters sampled from the prior. We then inferred the model parameters using the optimized *s*_abc_ of 200,000 and using the optimized *m* value. The accuracy was high for *R*_in_ (inversion rate) and *R*_tr_ (translocation rate): *r*^2^ of 0.962 and 0.973, respectively. The corresponding MSE scores were 0.007, 0.005, respectively. Moderate *r*^2^ values were obtained for *R*_fi_ (fission rate), *R*_fu_ (fusion rate), *a*-parameter, and *ch*_root_: *r*^2^ of 0.871, 0.841, 0.815, and 0.876, respectively. The corresponding MSE scores were 9.49e-6, 0.008, 0.0135, 1.933, respectively (fig. 7). We note that both the accuracy and the variance of the inferred model parameters may depend on their true value and on the assumed prior. For example, as the rate of fission and fusion events increases and approaches the boundaries of the assumed prior, the inference of these rates becomes slightly biased and less accurate (fig. 7).

#### Yeast Dataset Results

**Fig. 8.**
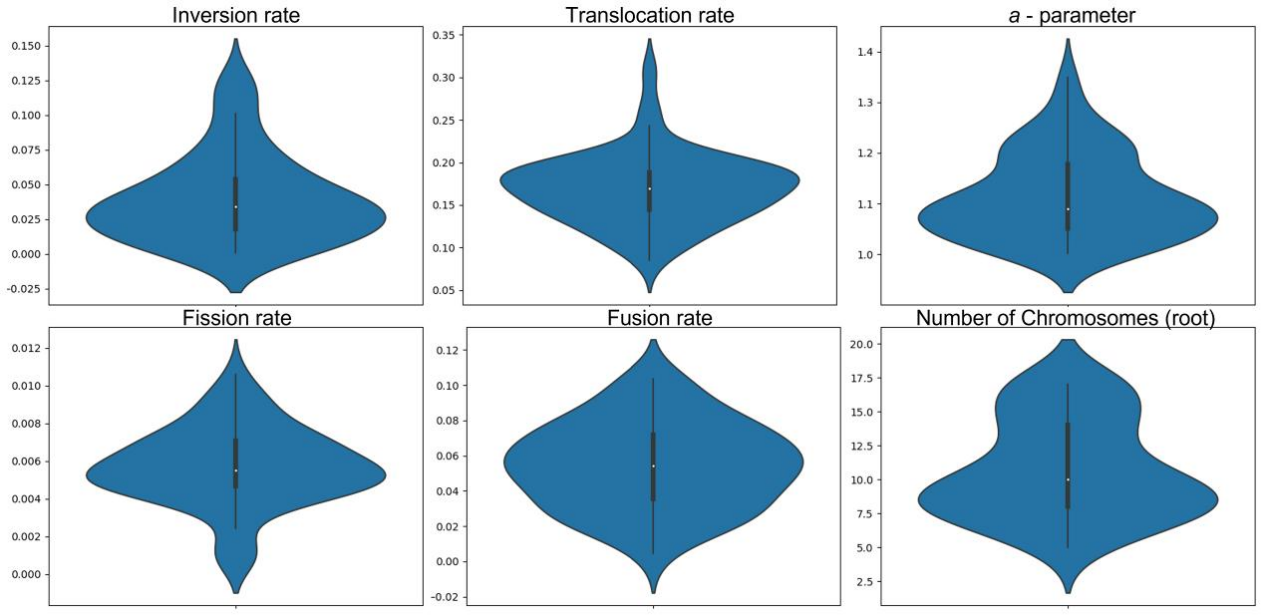
Violin plots of the 50 closest simulations for the yeast data inference. Note that although the prior was distributed uniformly in the ranges seen in the graphs, the posterior is clearly centered around a much smaller range of values for all parameters except for the root chromosome number.

We used the optimized *s*_abc_ and *m* values. The estimated parameters were 0.039, 0.17, 1.11, 10.74, 0.0545, 0.0057 for *R*_in_*, R*_tr_, *a*-parameter, *ch*_root_*, R*_fu_, *R*_fi_, respectively. We also note that the ABC posterior distributions, depicted from the 50 closest simulations, are mostly centered around a single value (fig. 8). These results suggest that in yeast, similar to the bacterial results, inversions are more common than translocations. The average block size was 8.14.

### Credible Regions

We next used simulations to quantify the accuracy of the ABC estimated posterior distributions. Ideally, each true parameter should be 90% of the times within its 90% ABC credible regions (the 90% ABC credible regions for each parameter is the range of the inferred parameter in the retained simulations, after discarding the top and bottom 5% of the values). Our results using the *M*_1_ model show that the ABC credible regions are moderately accurate for *R*_in_*, R*_tr_, *R*_fu_, *R*_fi_ (obtained fractions of 0.91, 0.89, 0.845, and 0.815, respectively). In contrast, the ABC credible regions are too narrow for the *a*-parameter and *ch*_root_ (obtained fraction of 0.675 and 0.72, respectively).

### ABC Compared to Parsimonious Method

We note that some of the presented summary statistics can be regarded as a part of a parsimonious inference scheme for the used parameters. One may suggest that even one of those summary statics may be sufficient to accurately inferring some of the parameters, thus making the ABC scheme unnecessary. We performed feature base selection by performing a pipeline similar to the one used for the ABC in which selection was done using a single summary statistic. For all features and all parameters, the *MSE* score for an inference using the ABC scheme with the full set of summary statistics was significantly better than the inference based on a single summary statistic (see supplementary table S2*a*and S2*b*, Supplementary Material online).

## Testing of Basic Assumptions

### The Sensitivity of the Inference Scheme to Inaccuracies in the Underlying Tree and the Assumptions of the Prior Distributions

#### Testing the Effect of Inaccuracies in the Phylogenetic Tree

Inference of phylogenetic trees is prone to inaccuracies and biases. Using different stochastic models or different sequences for the tree inference can result in changes in the branch lengths and the topology of the reconstructed tree. To test the sensitivity of our inference scheme to tree inference uncertainty, we created 10 “noisy” trees, generated by Jackknifing 500 positions from the multiple sequence alignment of the core genome, which was used for the inference of the original tree. Trees were then inferred based on this sample using the same procedure that was used to reconstruct the original tree (See Methods). All trees differ from the original tree in terms of topology (Robinson–Foulds distances that ranged from 2 to 10). This analysis clearly shows that inaccuracies in the inferred tree results in a significant reduction in performance for all model parameters (supplementary table S3, Supplementary Material online). Nevertheless, even when the Robinson–Foulds distance between the true and the noisy tree was 10 (meaning that in each tree there are five partitions that are absent in the corresponding tree), significant correlations were observed for all parameters except for the number of chromosomes in the root.

#### Testing Alternative Prior Distributions

The assumed prior distribution over the set of model parameters can affect the posterior distribution of the parameters and as a result, the inferred values. We assumed a uniform distribution over all model parameters, aiming to have uninformative priors. To test whether the choice of priors affects the inferred estimates of model parameters, we tested alternative prior distributions: Log-normal and Gamma distributions for the continuous parameters and the Poisson distribution for the discrete distributions. Assuming these alternative prior distributions had a minor effect on the inferred parameters, giving similar results to those inferred assuming the uniform priors. This was true both for the microbial and yeast data (Supplementary tables S4*a–*S4*d*, Supplementary Material online). For example, the number of ancestral chromosomes for the yeast data was estimated to be 10.74, 10.8, and 11.86 for the uniform, gamma, and log-normal priors, respectively. The parameters that seem most sensitive to the prior were the chromosome fission and fusion rates.

#### Relation Between the Scale Parameter (h) and the True Parameters

As explained in the Methods, when analyzing a dataset, *y*_obs_, the ABC method used in this work retains a fix number (*m*) of simulations. This is different from other implementations of the ABC approach, in which a predetermined distance cutoff (*h*) is used. In these other implementations either the number of retained simulations or the total number of simulations (*s*_abc_) is not fixed a-priori. This may result in very few retained simulations or, in contrast, if simulations are generated until a fixed number of simulations have distances lower than *h*, the running time of the ABC approach is unlimited. Our approach guarantees fixed running times by retaining a fixed number of simulations. In our implementation, retained simulation may have varying maximal distances between the vector of summary statistics of retained simulations and the summary statistics vector of *y*_obs_. In supplementary fig. S3, Supplementary Material online, we plot for the maximum distance obtained among the retained simulations against the true value of the model parameter used to generate the data. The results suggest that for most model parameter, the difficultly to generate simulations that resemble the analyzed dataset does not strongly correlate (*r*^2^ < 0.2) with the value of the parameter used to generate *y*_obs_. For two model parameters, a stronger dependence was observed. As the rate of the inversion rate parameter increases, it is more difficult to generate simulations that are similar to *y*_obs_. An opposite trend is observed for the translocation rate. Thus, when an empirical dataset is characterized by many inversions, it may be beneficial to increase *s*_abc_ when computational resources are available.

## Discussion

In this work, we developed two genome-rearrangement Markov models. In our basic model, a single chromosome is assumed and two order changing events are included: inversions and translocations. In the richer model, we allowed for multiple chromosome genomes and introduced changes in chromosome numbers via fission and fusion events. We showed that for both models, the ABC method can be used to accurately infer genome-rearrangement model parameters.

Both models used here assume that the extant species contain the same gene repertoire. Aiming at a more realistic model, the next step would be to allow gene-content-altering events, that is gene gains, for example, via gene duplications or lateral gene transfers, and gene losses. Enabling such events will require adjustments in the minimalistic genome format, as it is important to track all genes that belong to the same gene family when comparing two or more genomes. For example, assume that the ancestral genome was [1,2,3,4], one of the extant genomes is [1,2,3,4], but in the other extant genomes, gene 3 underwent duplication. We will differentiate the copies of gene 3 by naming them 3_a_ and 3_b_. The second extant genome may be [2,3_a_,1, −4, −3_b_]. We note that both copies of gene 3 participate in *UB*s, and thus we need the ability to identify both copies as gene 3.

Allowing changes in genome content among extant species will allow the analysis of entire genome contents in the inference process as opposed to core genomes used here. This may also result in a better inference of the model parameters considered here. For example, one source of error in the current implementation is that adjacent genes in the minimalistic genome format may be separated by several genes that are not part of the core genome. By accounting for these non-core genes, this source of error may be alleviated.

As mentioned above, chromosome-number changing events in our *M*_1_ model do not fully capture the underlying evolutionary dynamics. Events such as whole-genome duplications (Glasauer and Neuhauss 2014; Landis et al. 2018) are not represented in our current model. However, the proposed ABC scheme presented here may be used to infer whole-genome duplication events even after the genomic signature for such events was eroded by numerous ensuing genome-rearrangement events.

In order to simulate genomes with several chromosomes, one has to assign genes to a set of ancestral chromosomes. While the number of ancestral chromosomes was included as a parameter of *M*_1_, the size distribution of ancestral chromosomes was assumed to follow a multinomial distribution, that is each gene has the same probability to be assigned to each ancestral chromosome. A possible improvement would be to test several size distributions that may better reflect empirical chromosome sizes. We note that each set of model parameters dictates a stationary distribution of chromosome sizes. Thus, another possibility is to infer such a distribution from the model parameters and use it to sample chromosome sizes at the root. This will necessitate computing the stationary distribution for each set of model parameters drawn from the prior. Whether assuming that the stationarity distribution is justified for empirical datasets remains to be tested.

When simulating genomic data, one aims at mimicking the dynamics of evolution observed in empirical datasets. Given a genome as input data, we could use the described tool to extract the posterior distribution of the genome-rearrangement parameters.

The work presented here was motivated by the need to design a more realistic genome simulator. The idea behind this is that for a realistic simulation, one should aim to imitate empirical data. Current simulators, such as ALF (Dalquen et al. 2012), assume changes in the gene order, but without a statistical method to learn the rates of such events, one cannot guarantee that the simulation will provide a good representation of reality. Therefore, given an empirical dataset (or datasets) one could use the presented method to extract estimations for the posterior distribution of the genome rearrangement parameters. Similar tools can be used to extract other evolutionary parameters, for example SPARTA-ABC (Loewenthal et al. 2021) can be used to extract posterior distributions of parameters relevant for small insertions and deletions. These posterior distributions can be used to simulate an entire genome data, which will better reflect empirical datasets compared to data for which the parameters were drawn randomly. Such simulators can be highly beneficial, for example when comparing genome-alignment algorithms (Marçais et al. 2018; Armstrong et al. 2019; Krasheninnikova et al. 2020).

In this work, branch lengths were optimized by analyzing substitution events rather than directly from gene order data. Furthermore, all model parameters were normalized to the number of substitutions. The justification behind this, is that genomic rearrangement events, substitution events, and time of divergence are all expected to be correlated. However, it may be that two species have recently diverged, and their genomes differ by a small number of substitutions, yet, multiple genomic rearrangement events have occurred between them, possibly contributing to the separation between these species. In such a case, the underlying assumptions of our model would fail. To account for such cases, one possibility would be to estimate branch lengths directly in terms of rearrangement events from the gene order data. In such a case, a free model parameter would be designated for each type of event for each branch. This will entail many more parameters that need to be inferred within the ABC framework and may lead to an over-parameterized model. Another option would be to assume that the ratio between all types of events is the same across all branches, and only the total number of rearrangement events varies among lineages. This will reduce the number of free parameters, but still, it will result in roughly 2n additional free parameters in the inference scheme, where *n* denotes the number of analyzed species. Yet another option would be to construct priors for the number of rearrangement events based on the number of substitutions. Clearly, model selection among these alternatives is an interesting research direction.

The model presented here infers genome rearrangement events based on gene order data. Each coding gene is a block, and its size in base pairs is ignored. Non-coding regions are ignored as well. A model that accounts for the genomic DNA sequence, non-coding regions, and gene lengths will be more realistic. However, modeling genome rearrangements on the DNA level has several inherent caveats: 1) Non-coding DNA sequences contain more “noise”, that is, they contain a higher number of small-scale changes such as point mutations and insertions and deletions of small segments. Thus, comparing or aligning of non-coding areas among genomes is harder and prone to higher levels of uncertainty; 2) simulating the entire genome sequence is computationally heavy. For example, bacterial genome size is on the scale of millions of base pairs but contains only around 5,000 genes (Land et al. 2015). Inference on the DNA level would make the genome simulation and the extraction of summary statistics from genomes, a much more computationally challenging task.

When inferring empirical data, we rely on data created with other tools. The data may be prone to noise and inaccuracies that result from these tools. For example, phylogenetic trees may have different topology or branch lengths, depending on the data and tools used to infer them. Genes may be disregarded due to identification or sequencing errors or mislocated due to scaffolding errors. Errors in the inference of orthology relationships can also be a source of inaccuracies in the inference of rearrangement events. We hypothesize that for the curated empirical datasets used in this study, the impact of these factors is negligible. However, caution is needed when analyzing draft genomes with low coverage.

Despite these limitations, our work is valuable and can lead to insights from the ever growing available genomic datasets. An example for such data set is the “Genome 10K” project, which aims at obtaining at least one genome from each vertebrate genus (Koepfli et al. 2015). The data collected in their project, along with the methodology described here, can help elucidate the dynamics of genome rearrangements and their effect on speciation.

## Methods

### Microbial Data

For the *M*_0­_ model, we used a microbial dataset as a case study. We established an *Escherichia* species dataset containing 73 genomes (62 *Escherichia coli*, 10 *Shigella*, and 1 *Escherichia fergusonii*), extracted all open reading frames in each genome, detected ortholog groups, and reconstructed their phylogeny using M1CR0B1AL1Z3R (Avram et al. 2019). Next, we filtered out all ortholog groups that were not classified as core genes, that is not present in all 73 extant species, resulting in a total of 1,875 core ortholog groups. We applied over these 1,875 (core) groups an in-house Python script to convert their DNA sequences into the minimalistic format as follows: each orthologs group was labeled with a unique number (from 1 to 1,875) and the sequences of its group members were replaced by this corresponding number with a sign (+/−) that corresponds to its direction. Once we obtained the minimalistic format, we used it in the inference process described above. Genome data in the minimalistic format, as well as the phylogenetic tree file and the in-house script are available in the project repository.

### Yeast Data

For the *M*_1­_ model, we used a yeast dataset as a case study. We collected genome data of 18 *Candida* species from the *Candida* Gene Order Browser (CGOB) (Maguire et al. 2013). We used in-house scripts to translate the CGOB alignment file (named “Pillars”) to our minimalistic format. As our models assume the same genome content for all genomes, we reduced the genomes to core genomes with a size of 2,714 genes. The original data, the minimalistic format file, the scripts, as well as the tree file, are available in the project repository.

## Supplementary Material

msac231_Supplementary_Data

## Data Availability

The analyzed data as well as the in-house scripts used for this study can be found at: https://github.com/asher-616/Genome-Rearrangement-ABC.
