# Supplementary material for: An Approximate Bayesian Computation Approach for Modeling Genome Rearrangements
Source: Mol Biol Evol. 2022 Oct 25;39(11):msac231. doi: 10.1093/molbev/msac231 (PMC9692237; doi:10.1093/molbev/msac231)
Supplement: msac231_Supplementary_Data [file msac231_supplementary_data.pdf]

Supplementary Information

An approximate Bayesian Computation Approach for Modeling Genome Rearrangements

Asher Moshe[1], Elya Wygoda[1], Noa Ecker[1], Gil Loewenthal[1], Oren Avram[1], Omer Israeli[1], Einat Hazkani-Covo[2], Itsik Pe'er[3], and Tal Pupko[1†]

[1]The Shmunis School of Biomedicine and Cancer Research, George S. Wise Faculty of Life Sciences, Tel Aviv University, Tel Aviv 69978, Israel.

[2]Department of Natural and Life Sciences, Open University of Israel, Ra'anana, Israel.

[3] Department of Computer Science, Columbia University, New York, New York, USA.

† To whom correspondence should be addressed:

Tal Pupko, Tel: +972 3 640 7693; Fax: +972 3 642 2046; E-mail: talp@tauex.tau.ac.il

Keywords: Genome rearrangement, Approximate Bayesian computation, Genome evolution.

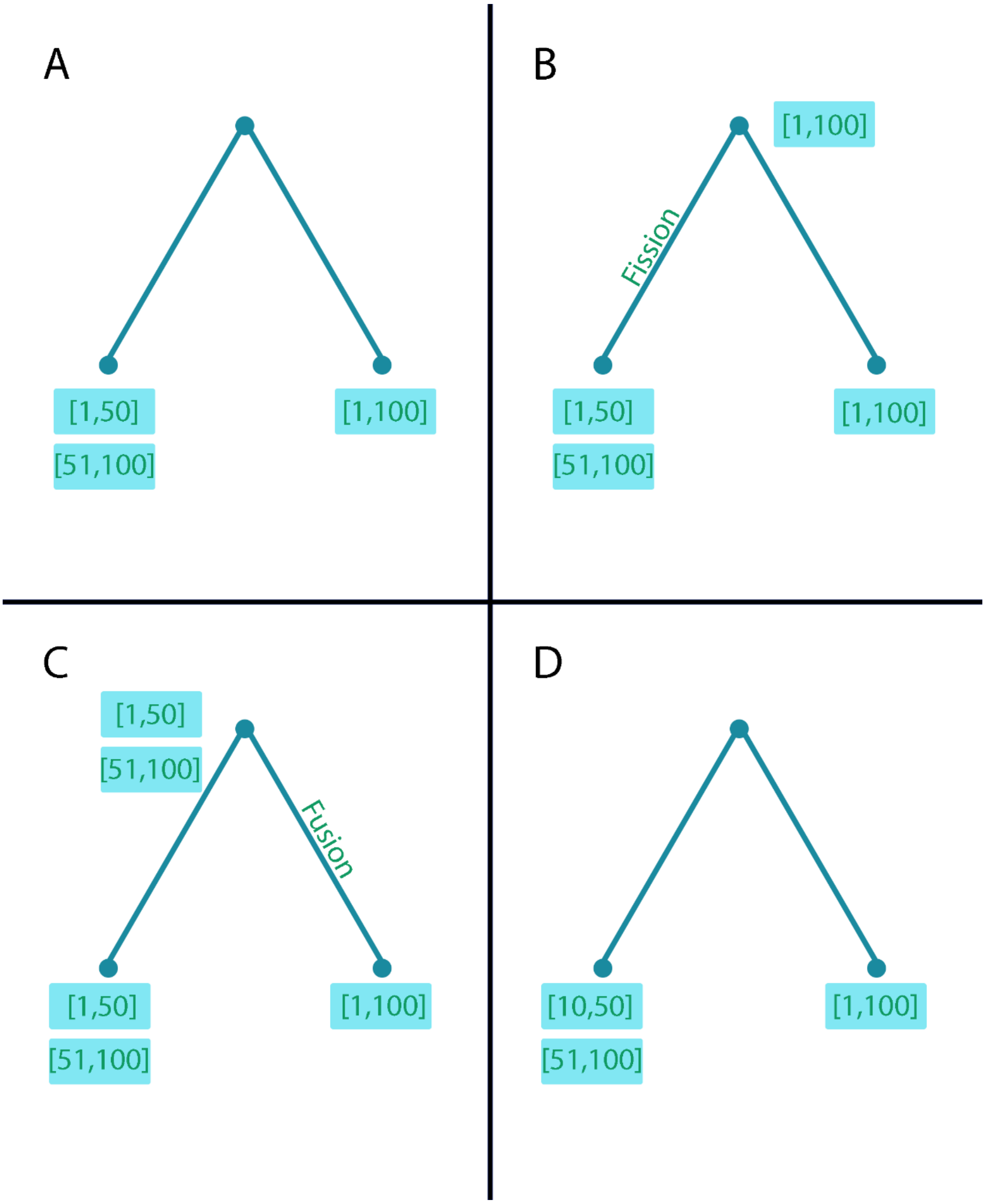

Fig. S1: An example of a probable fission-fusion (PFF) event. (A) An example tree with two species. The left one has two chromosomes. The first starts with gene 1 and ends with gene 50, and the second starts with gene 51 and ends with gene 100. The right species has a single chromosome that starts with gene 1 and ends with gene 100; (B) One possible scenario that could have generated these data is a fission event in the lineage leading to the left species; (C) The same data could have been generated by a fusion even in the lineage leading to the right species; (D) An example of data that do not count as a PFF. For data to be considered as a PFF, both tips of the minimal chromosome (MC) of the right species, i.e., both 1 and 100, should be present as tips in the set of MCs of the left species. However, gene 1 does not satisfy this condition.

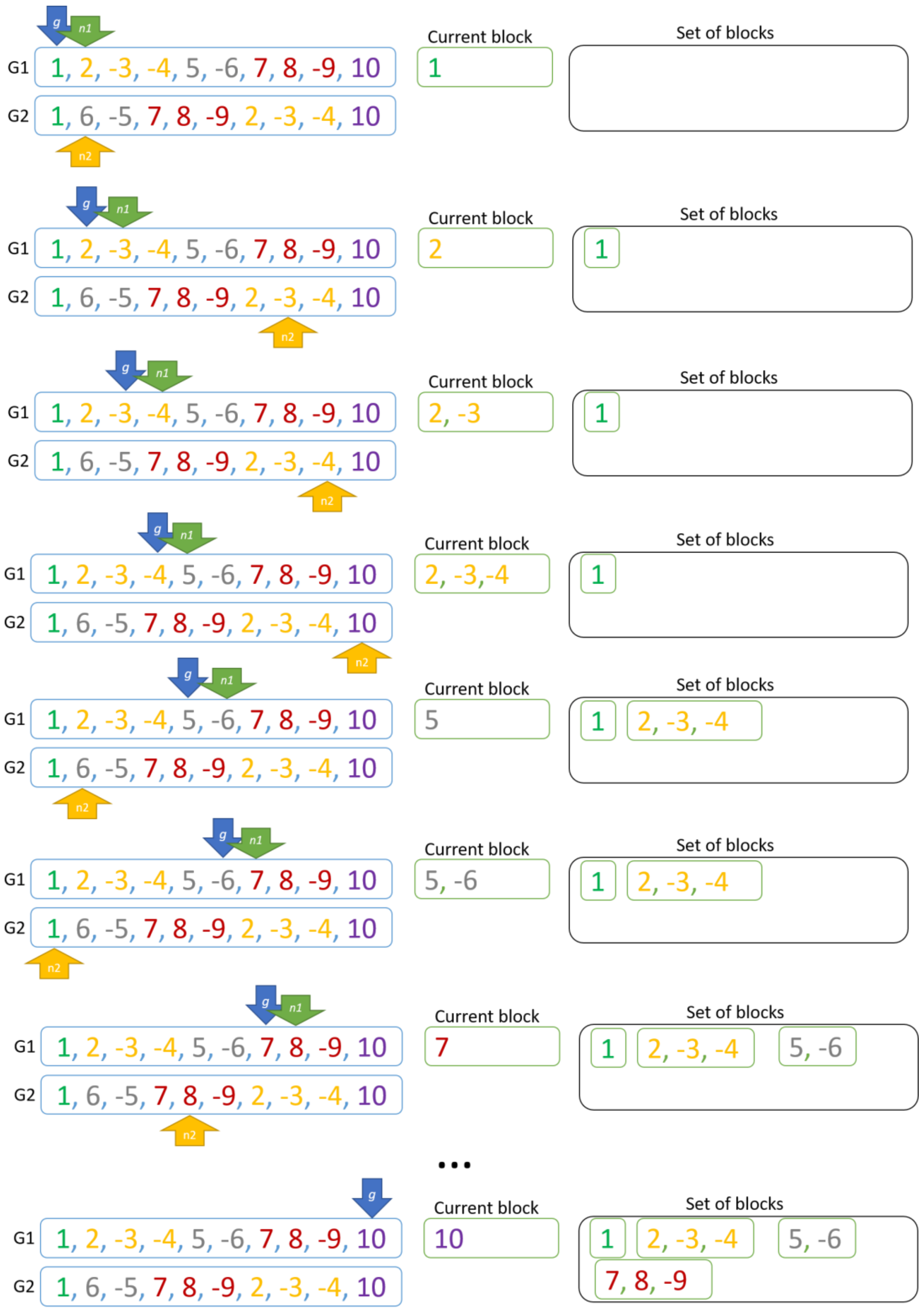

Fig. S2. Demonstration of the unique-blocks algorithm. We start with $g \coloneqq 1$, $n_1 \coloneqq 2$. We locate gene 1 in the second genome, and we compute the direction: $d = 1$, suggesting that we must search the next gene in the second genome to the right of gene $g = 1$. Thus, $n_2 \coloneqq 6$. We end the first block, which is of size one and contains only gene 1. We then move to $g \coloneqq 2$, $n_1 \coloneqq -3$. We start a new block that contains $g = 2$. The gene g is with a positive sign both in G_1 and G_2, and hence $d = 1$. To find n_2 we hence search in the right direction, and thus $n_2 \coloneqq -3$. In this case, $n_1 = n_2 d$, and therefore, we assign $g \coloneqq -3$ and append gene -3 to the current block. We continue this scanning process until we reach the end of G_1.

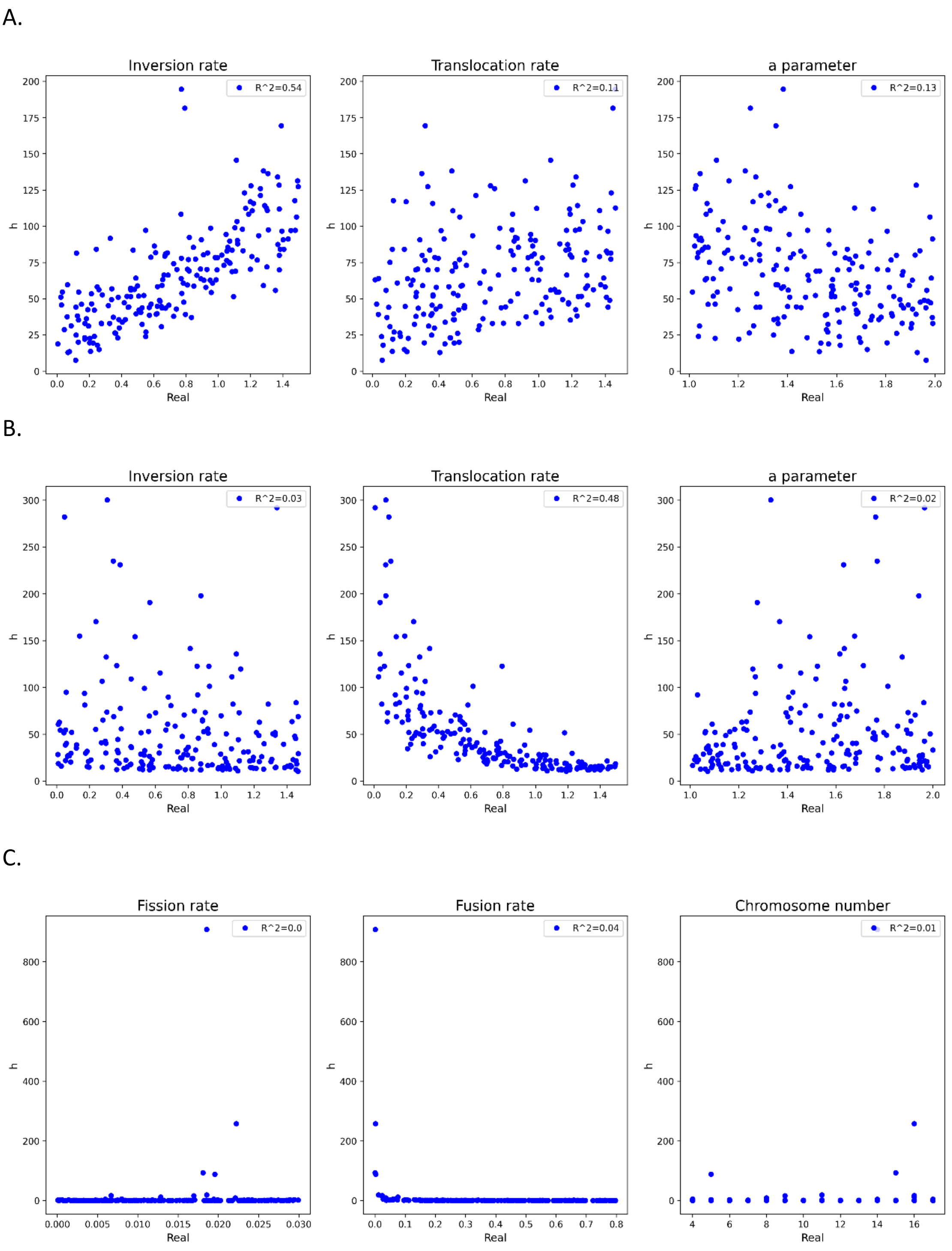

Fig S3. Correlation between model parameters and the scale parameter (h) for the simulation based result. A: M0 and microbial data. B: M0 and yeast data. C: M1 and yeast data.

Table S1: optimization of the number of retained simulations. Each row corresponds to a different number of retained simulations within the ABC inference scheme (specified by the value of m). The accuracy for each parameter and each value of m is measured by the coefficient of determination, r^2 (top table) and MSE (bottom table). The different parameters are specified in Table 1.

m	a- parameter	R_{in}	R_{tr}	ch_{root}	R_{fu}	R_{fi}
**1**	0.752	0.948	0.967	0.789	0.632	0.762
**2**	0.809	0.956	0.969	0.826	0.732	0.818
**5**	0.836	0.963	0.975	0.861	0.788	0.858
**10**	0.842	0.971	0.978	0.858	0.812	0.875
**20**	0.843	0.968	0.977	0.848	0.808	0.866
**50**	0.833	0.966	0.975	0.831	0.816	0.87
**100**	0.811	0.962	0.972	0.806	0.802	0.863
**200**	0.782	0.956	0.967	0.782	0.786	0.854
**500**	0.707	0.941	0.957	0.735	0.76	0.838
**1,000**	0.621	0.925	0.946	0.688	0.733	0.82
**2,000**	0.51	0.903	0.933	0.626	0.697	0.797

m	a- parameter	R_{in}	R_{tr}	ch_{root}	R_{fu}	R_{fi}
**1**	0.0206	0.00915	0.00624	3.28	0.0184	1.74E-05
**2**	0.0159	0.00779	0.00589	2.69	0.0134	1.33E-05
**5**	0.0137	0.00653	0.00474	2.15	0.0106	1.04E-05
**10**	0.0132	0.00512	0.00413	2.2	0.0094	9.14E-06
**20**	0.0131	0.00561	0.00437	2.35	0.00959	9.78E-06
**50**	0.0139	0.00601	0.00478	2.62	0.00918	9.53E-06
**100**	0.0157	0.00669	0.00533	3.0	0.00989	1.00E-05
**200**	0.0181	0.0078	0.00628	3.37	0.0107	1.06E-05
**500**	0.0243	0.0105	0.00811	4.1	0.012	1.18E-05
**1,000**	0.0314	0.0134	0.0101	4.84	0.0133	1.32E-05
**2,000**	0.0407	0.0173	0.0127	5.8	0.0152	1.48E-05

Table S2: A comparison between inference using each single summary statistic to inference using the full ABC scheme for summary statistics and parameters. A: M_0 model, B: M_1 model

A.

Summary statistics	MSE		
	R_{in}	R_{tr}	*a*-parameter
Full ABC	0.007	0.005	0.0135
Number of *UBs* of size 1	0.117	0.154	0.0793
Number of *UBs* of size 2	0.143	0.081	0.0841
Number of *UBs* of size 3	0.171	0.0411	0.0869
Number of *UBs* of size 4	0.155	0.0422	0.0884
Number of *UBs* of size 5	0.146	0.047	0.09
Number of *UBs* of size 6	0.144	0.0523	0.09
Number of *UBs* of size 7	0.144	0.0595	0.092
Number of *UBs* of size 8	0.141	0.0735	0.091
Number of *UBs* of size 9	0.151	0.0725	0.0913
Number of *UBs* of size 10	0.145	0.0883	0.0914
Number of *UBs* larger than 10	0.12	0.116	0.0811
Number of *PIUBs* of size 1	0.124	0.0923	0.0973
Number of *PIUBs* of size 2	0.145	0.0465	0.0875
Number of *PIUBs* of size 3	0.153	0.0295	0.0884
Number of *PIUBs* of size 4	0.154	0.0271	0.093
Number of *PIUBs* of size 5	0.149	0.0342	0.092
Number of *PIUBs* of size 6	0.155	0.0484	0.0923
Number of *PIUBs* of size 7	0.168	0.0673	0.0915
Number of *PIUBs* of size 8	0.161	0.0794	0.0891
Number of *PIUBs* of size 9	0.161	0.0799	0.0886
Number of *PIUBs* of size 10	0.167	0.0984	*0.0899*
Number of *PIUBs* larger than 10	0.158	0.0834	*0.0881*

B.

Summary statistics	MSE		
	Ch_{root}	R_{fu}	R_{fi}
Full ABC	1.933	0.008	9.49e-06
Minimum chromosome number in a leaf	12.595	0.0358	*4.16e-05*
Maximum chromosome number in a leaf	12.934	0.0255	4.501e-05
Mean chromosome number in the leaves	14.89	0.0233	2.927e-05
Variance of chromosome number in the leaves	12.739	0.0289	6.588e-05
Size of set of *MC*s in the root	14.78	0.0257	2.207e-05
Size of Tip set at the root	14.728	0.0235	2.68e-05
Number of unique tips	15.026	0.025	*2.246e-05*
Number of *PFF*s	14.814	0.021	*7.279e-05*

Table S3: Sensitivity of the ABC methodology to inaccuracies of the phylogenetic tree. For each of the 10 erroneous tree, 100 executions of the methodology were performed. The "original" tree is the yeast-based phylogeny (see Methods). The different parameters are specified in Table 1. A: The r^2 between the simulated and inferred parameters. B: The MSE between the simulated and inferred parameters.

A.

Tree	a- parameter	R_{in}	R_{tr}	ch_{root}	R_{fu}	R_{fi}	Robinson-Foulds
original	0.843	0.952	0.976	0.881	0.847	0.886	0
1	0.630	0.867	0.623	<0	0.376	0.487	6
2	0.628	0.564	0.561	0.631	0.475	0.621	4
3	<0	0.448	0.887	0.873	0.683	0.718	8
4	0.608	0.863	0.553	<0	0.543	0.668	10
5	0.640	0.854	0.515	0.038	0.363	0.507	6
6	0.669	0.816	0.763	0.104	0.476	0.716	10
7	0.590	0.682	0.368	0.032	0.471	0.655	4
8	0.563	0.783	0.453	0.131	0.467	0.625	4
9	<0	0.259	0.772	0.879	0.681	0.677	2
10	0.700	0.654	0.661	0.164	0.213	0.446	6

B.

Tree	a- parameter	R_{in}	R_{tr}	ch_{root}	R_{fu}	R_{fi}	Robinson-Foulds
original	0.012	0.0094	0.0046	1.9	0.008	9.4E-6	0
1	0.029	0.026	0.074	16.34	0.035	4.22e-5	6
2	0.029	0.086	0.086	5.89	0.029	3.12e-5	4
3	0.104	0.109	0.022	2.03	0.018	2.32e-5	8
4	0.031	0.027	0.088	16.3	0.026	2.73e-5	10
5	0.028	0.029	0.095	15.3	0.036	4.06e-5	6
6	0.026	0.036	0.046	14.3	0.03	2.34e-5	10
7	0.032	0.063	0.124	15.5	0.03	2.84e-5	4
8	0.034	0.043	0.107	13.9	0.03	3.09e-5	4
9	0.108	0.068	0.15	1.94	0.018	2.66e-5	2
10	0.024	0.068	0.066	13.3	0.044	4.6E-5	6

Table S4. The effect of different prior distributions. A: Assumed priors for M_0. B. Assumed priors for M_1. C. The effect of different prior distributions over the estimated parameters for the microbial empiric data. D. The effect of different prior distributions over the estimated parameters for the yeast empiric data.

A.

Parameter	prior	
	Log-normal	Gamma
R_{in}	LN(-0.78,1)	Γ(2.0,0.375)
R_{tr}	LN(-0.78,1)	Γ(2.0,0.375)
a- param	Un(1.001,2.0)	Un(1.001,2.0)

B.

Parameter	prior	
	Log-normal	Gamma
R_{in}	LN (-0.78,1)	Γ (2.0,0.375)
R_{tr}	LN (-0.78,1)	Γ (2.0,0.375)
a- param	Un (1.001,2.0)	Un (1.001,2.0)
R_{fi}	LN (-4.7,1)	Γ (2.0,0.0075)
R_{fu}	LN (-1.4,1)	Γ (1.75,0.229)
Ch_{root}	Poisson (11)	Poisson (11)

C.

Prior	Inversion	Translocation	a parameter
Uniform	0. 342	0. 077	1.09
Gamma	0.295	0.090	1.06
Log-normal	0.121	0.102	1.482

D.

Prior	Inversion	Translocation	a parameter	Chr num	fission	fusion
Uniform	0.039	0.17	1.229	10.74	0.0057	0.0545
Gamma	0.0706	0.157	1.171	10.8	0.00595	0.058
Log-normal	0.0688	0.143	1.11	11.86	0.011	0.26
